# Replication After Collins: An Ethnography of Current Replication Studies in Psychology

**DOI:** 10.1177/03063127251382558

**Published:** 2025-10-26

**Authors:** Jonna Brenninkmeijer, Maarten Derksen, Stephanie Meirmans, Jeannette Pols

**Affiliations:** 1 Amsterdam UMC, University of Amsterdam, Amsterdam, The Netherlands; 2 University of Groningen, Groningen, The Netherlands

**Keywords:** replication, psychology, ethnography, tacit knowledge, sociology of scientific knowledge

## Abstract

Ethnography and other empirical studies of replication played a significant role in the sociology of scientific knowledge (SSK) during the 1970s and 1980s. Collins and other proponents of SSK highlighted that exact replication was impossible, knowledge was often tacit and hard to explicate, and that results were always socially negotiated. We revisit these observations and compare them with our ethnography of current replication practices in psychology. We highlight the diversity of negotiations, tacit knowledge, and practices surrounding replication more generally. We also examine how replication encourages and necessitates reflection on the research process—on what should count as the same experiment and the same result, on the role of tacit knowledge (such as skill, experience, judgment, and feeling) in science, and on what replication entails and whether it is worth the effort. Researchers who conduct replications can be seen as ethnographers of their science, reflecting on the scientific process and the challenges of producing results.

Ethnographic and other empirical studies of scientific replication were an important part of the new sociology of science during the 1970s and early 1980s. For Collins, Pinch, Gilbert, Mulkay, Knorr Cetina, and other proponents of the sociology of scientific knowledge (SSK), these studies supported their argument that knowledge is fundamentally social and that philosophy paints an inadequate picture of science. They particularly took issue with Popper’s claims (e.g. Popper, 2002, p. 45) that replication is an essential part of scientific research: ‘Only when certain events recur in accordance with rules or regularities, as in the case of repeatable experiments, can our observations be tested—in principle—by anyone .… Only by such repetition can we convince ourselves that we are not dealing with a mere isolated “coincidence”, but with events which, on account of their regularity and reproducibility, are in principle inter-subjectively testable.’ However, according to the new generation of sociologists of science, this was not how science worked.

Collins, especially, put considerable effort into debunking this Popperian view of replication and science in general. Based on several empirical studies of replication practices in various scientific laboratories—presented in a series of papers and culminating with his 1985 book *Changing Order*—H. M. Collins (1985) developed a radically different conception of replication and scientific inference. His studies of replication in practice became the central support for his ‘empirical programme of relativism’ (H. M. Collins, 1981b). According to Collins, replication in practice showed the interpretative flexibility of empirical claims. Empirical observations, such as the results of a replication study, always allow multiple interpretations and thus multiple inferences. Collins concluded that it is not reality that determines which interpretation prevails, but rather social factors. The controversies that typically followed such replication attempts provided a rich source of material in support of this argument. ‘The salience of alternative interpretations of evidence, which typifies controversies, has acted as a lever to elicit the essentially cultural nature of the local boundaries of scientific legitimacy normally elusive or concealed’ (H. M. Collins, 1981b, p. 4).

On several occasions, Collins claimed that ‘the socially-negotiated nature of experimental replication’ is itself an outcome that has been replicated in multiple sociological studies of replication (H. M. Collins, 1981b, p. 4, 1982). Both Mulkay (1984) and Ashmore (1988) have discussed the reflexive implications of this claim. They argue that if replication is socially negotiated, then this very conclusion should also be the product of social processes—among sociologists of science—and, in principle, subject to renegotiation. Ashmore pointed out the paradox that this creates: Negotiating the negotiated nature of replication simultaneously affirms this finding and relativizes its status as a ‘finding’. Collins has rejected this reflexive move and its paradoxical implication by stipulating that sociologists of science, just like the scientists they study, should maintain a realist perspective with respect to their object of study and ignore the social processes of their practice (H. M. Collins, 1981a).

While this discussion is undoubtedly fascinating, reducing sociological studies of replication to the single conclusion that replication is socially negotiated oversimplifies their findings. The value of the work of Collins and his colleagues lies equally in the empirical detail with which they have ‘documented’ this view of replication and of scientific knowledge more generally (H. M. Collins, 1981b, p. 4). Although ‘SSK’ and the ‘empirical programme of relativism’ may have declined in influence as schools or touchstones in science and technology studies (STS), the studies of replication they inspired remain valuable—not only for their earlier role in theoretical debates, but also for their practical relevance. These studies are not only useful as levers to uncover interpretative flexibility, but also as explorations of replication in practice, the foundation that we seek to build this work upon.

In this article, we revisit these studies of replication and compare their observations with those from our study of current replication practices. Our focus is not on the larger epistemological issues that were important to Collins and his colleagues—relativism, constructivism, and reflexivity—but on the observations that buttressed their positions on these matters. We have two reasons to engage in this comparison. First, science is not static, and scientific practices may change over time. Few empirical studies of replication have been conducted in STS since the mid-1980s. It is quite possible that what held true in replication studies in biochemistry during the 1970s no longer applies today, and the same goes for other fields. The changing nature of science is particularly evident when it concerns replication. In several disciplines, a ‘replication crisis’ was declared in the early 2010s, prompted among other factors by failures to reproduce the results of high-profile studies (e.g., Begley & Ioannidis, 2015; Pashler & Harris, 2012). The replication studies that we have followed were conducted in psychology, where a reform movement has emerged that places replication at its core. To remedy the loss of confidence in the discipline’s existing claims, it is argued that replication should become an essential part of the psychologists’ epistemic practices, and the results of replication studies, including null results, should be published (see, e.g., Zwaan et al., 2018).

A second reason to compare the observations of the older sociological studies of replication with our own is the diversity of science. The sociological studies of replication during the 1970s and 1980s were mostly concerned with natural sciences (but see H. M. Collins & Pinch, 1979, 1982, for a discussion of parapsychology). Psychology is a discipline that often aspires to the status of natural science, but has a somewhat distinct character. Although many psychologists model their research on natural sciences, experiments involving human beings pose distinct practical and ethical problems. Moreover, many psychologists assign a unique nature to their object of study that distinguishes it from those of the natural sciences. Given the historical and cross-disciplinary diversity in science, it is worthwhile to extend the sociological studies of replication conducted during the 1970s and 1980s to the current time and another scientific field. In what follows, we first summarize the main insights of the sociological studies of replication during the 1970s and 1980s. Then, we introduce our study of replication and compare our observations to these earlier studies. To conclude, we summarize the results of the comparison and return to the issue of replication and reflexivity in STS.

## SSK on replication

In this section, we present a summary—inevitably partial—of the ideas that sociologists of science, and Collins in particular, developed about replication during the 1970s and 1980s. We focus on Collins’s work because he was then (and later) the most prolific author on this topic, but we note where other scholars supported or challenged his claims. Collins’s work on replication remains influential, and we briefly discuss several recent engagements with his ideas.

Collins began with the premise that the repeatability of observations is a fundamental requirement in science. He presented it as an undisputed fact—though, without providing evidence—that replicability functions as ‘the Supreme Court of the scientific system’ (H. M. Collins, 1985, p. 19). More fundamentally, he argued (p. 8) that perception in general is tied to regularity: ‘Perception and stability of perception are the same thing.’ Our concepts refer to objects and phenomena that possess some sense of stability and do not randomly appear, disappear, or change. Therefore, we simply cannot imagine a science that does not strive for the repeatability of its results (H. M. Collins, 1991).

Collins noted that, despite this fundamental requirement of repeatability, replication was seldom practised in science. Studies that copy the procedures of an earlier study—what are now often called ‘direct replications’—are rare (H. M. Collins, 1985). Similarly, the biochemists interviewed by Mulkay and Gilbert (1986) viewed what they referred to as ‘mere replications’ as rather disreputable. Likewise, Knorr (1979, p. 374) observed that scientists aimed for ‘distinctiveness’. Collins and Knorr connected this fact to the reward system in science, in which credit accrues to researchers with new results and innovative theories, rather than confirmations of other people’s work. Mulkay and Gilbert (1986) noted that the replicability of most findings is simply taken for granted, so doing replication studies is looked down upon, and a mere replication is not publishable (Mulkay, 1984). Scientists only attempt direct replications when there is reason to doubt the original results (H. M. Collins, 1985; Mulkay & Gilbert, 1986). For Mulkay and Gilbert’s (1986) biochemists, such replications aimed to identify the reason behind the production of questionable results.

Collins’s work focused on the issues that arise when replications are attempted. Two experiments are simultaneously alike and different in indefinitely many ways. Each has an indefinitely large number of characteristics that may or may not correspond to those of another experiment. Therefore, it is impossible to define a replication through a finite number of instructions—such as those contained in the methods section of a journal article. Although scientists and philosophers such as Popper conceive of replication as following a recipe, such an ‘algorithmic model’ of replication, as H. M. Collins (1975) termed it, does not work. Learning how to conduct an experiment is not a matter of learning a set of instructions; it is more like becoming part of a culture—one that defines what is and is not relevant in the experiment, without necessarily making this explicit or even consciously recognized. According to H. M. Collins (1975), this ‘enculturational model’ of knowledge is empirically more adequate than the algorithmic model, as it fits better with what he and others observed in replication studies.

The enculturational model implies that scientific knowledge, including the knowledge required to replicate an experiment, is to an important extent composed of tacit knowledge and tacit skill. It is only when problems occur or when teaching someone how to run an experiment, that an effort is made to make the tacit explicit. When there is no consensus about what the outcome of the experiment should be, the situation is even more complicated. These are the cases in which direct replication is simultaneously most useful and most difficult.

Researchers may be confronted with what H. M. Collins (1985) dubbed the ‘experimenters’ regress’: The correct outcome of an experiment depends on whether the predicted phenomenon exists. To determine whether it exists, we must conduct an experiment. Yet we cannot know whether the experiment was competently performed until we know the correct outcome. There is no independent criterion available to judge when the experiment has been conducted competently. There may be reasons to believe that the experiment is a valid way of producing and measuring the phenomenon. However, not only do such reasons always rely partially on tacit knowledge, but their applicability to this purportedly new phenomenon depends on the characteristics of this phenomenon, which are still under study and not yet agreed upon.^1^

Based on the experimenters’ regress and the role of tacit knowledge in science, Collins concluded that debates on the existence of a phenomenon can be resolved only through social processes. As such, the debates about the existence of the phenomenon and the right way to conduct the experiment become ‘congruent social processes … the social embodiment of the experimenters’ regress.’ (H. M. Collins, 1985, p. 89). He referred to these social processes as ‘negotiations’—rhetorical strategies may play a role, power and prestige may tip the balance, and friendships and enmities may come into play.

Mulkay and Gilbert (1986) noted that, although researchers are generally uninterested in ‘mere replication’, they often repeat each other’s experiments using different instruments or procedures, and to achieve starting points for further research. These researchers have a wider conception of replication, which includes variations on the original experiment (see also, Franklin & Howson, 1984).

The diversity of replication has also been noted by the STS scholars who have engaged with the recent replication crisis. For example, Feest (2016, 2019) argues that Collins sees replication controversies as implicitly revolving around the question of a phenomenon’s existence. However, in her view, replication studies are best approached as explorations of a phenomenon’s nature—exploration is more important than existence especially in psychology because of its ‘high degree of epistemic uncertainty and conceptual openness’. Through successive replications-with-variations, researchers can gradually flesh out what is relevant in the experiment and how the variables under study must be operationalized, a process that she calls ‘operational analysis’. The way that concepts should be operationalized tends to be much more uncertain in psychology than in the natural sciences. Peterson and Panofsky (2021) distinguish two motivations for replication studies: diagnosis and integration. In diagnostic replications—the type on which Collins focused—researchers aim to assess the truth of claims. In contrast, integrative replications are conducted with an eye on incorporating the original results into one’s research. This latter type of replication is quite common. Similarly, Guttinger (2019, p. 454) claims that replication appears rare only if one focuses exclusively on dedicated replication studies, while his so-called ‘micro-replications’, which are integrated into regular research practice, occur frequently.

Framing replication primarily as a means to determine the existence of a phenomenon has also led to fruitless controversies—particularly within psychology. Peterson and Panofsky (2021) warn that in a discipline with such high ‘task uncertainty’, diagnostic replications can create professional tensions (p. 601). There may be fundamental limits to diagnostic replication in psychology, given the unique historical, social, and cultural nature of its study object. For example, Peterson (2015) maintains that producing and stabilizing phenomena—a basic epistemic activity in many natural sciences—is often impossible in psychology, where phenomena are contextually variable. This has long been a topic of discussion in psychology, with some psychologists saying that variability makes direct replications of little use in psychology (e.g., Crandall & Sherman, 2016; Stroebe & Strack, 2014). However, Collins rejected a special status for psychology or the social sciences with respect to replication. He maintained that ‘science deals with observation, and observations must be stable … and therefore repeatable.’ (H. Collins, 2016, p. 78) He reluctantly admitted that the central role of language in human behaviour and society complicates replication, but nevertheless it must remain the aim. If psychology strives to be a science rather than an art replicability must be its ‘aspiration’ (H. Collins, 2016, p. 80). However, for current STS scholars, this stance ignores the diversity of science (e.g., Leonelli, 2018; Penders et al., 2019; Peterson & Panofsky, 2021).

## Methods and material

We analyse the motives behind, and the navigation of practical and methodological complexities in, replication studies in psychology. To do this we followed 16 replication studies, using interview and observation methods. These studies were a part of our broader project, *Replication in action*, in which we approached all 24 replication researchers—18 from the social sciences, five from medical science, and one from humanities—who were funded by the Dutch Research Council (NWO) and the Netherlands Organization for Health Research and Development (ZonMw) through a special Replication Studies programme conducted over three rounds between 2017 and 2019.^2^ Sixteen out of 18 replication researchers in social science agreed to participate in this study. Although they represent a variety of subfields—including clinical psychology, economic sociology/psychology, neuropsychology, pedagogy, and methodology—as well as diverse types of experiments, for the sake of readability we refer to all these studies as ‘psychology’. All these psychology replication studies were conducted by research teams of one or more principal investigators (PIs), PhD students, or postdoctoral researchers, and sometimes research assistants and methodologists. Some studies were conducted across multiple labs—both in The Netherlands and abroad—and involved additional research teams at those sites. We conducted semi-structured interviews, in Dutch or English, with all 16 PIs as well as approximately 20 other researchers involved in the studies—including PhD students, postdoctoral researches, and methodologists.^3^ Our interview guide contained questions about the original study and the replication process, expected results and impact, and the importance of replication studies. In six ongoing experiments, we conducted observations of research practices (e.g., research meetings, experiments, and experimental preparations), carried out follow up interviews, and we collected projects-related documents such as emails, protocols, reviews, proposals, and draft papers of most projects. This resulted in about 200 documents for the psychology studies, which JB analysed using ATLAS.ti. The analyses employed both deductive themes (e.g., motivation, meaning of replication results, tactics, and barriers to good replication) and inductive themes (e.g., context, psychology of replication, and reflection), using a total of 130 codewords. For this article, we focused on the codes or quotes most relevant to the work of Collins and colleagues (e.g., exactness, algorithmic model, tacit knowledge, experimenters’ regress, negotiation, and exploration). We employed an iterative approach, comparing insights from the literature with our empirical material. We identify quoted researchers in this article by pseudonyms and anonymize personal information.^4^

## Reasons for replication

One of the best-known findings of the sociological studies of replication in science is that researchers rarely copy another study’s experimental protocol—and only do so when they doubt the original results. According to the literature, replication still seems to be uncommon but is especially motivated by doubt about the original results or more generally by the alleged replication crisis (Baker, 2015; Clarke et al., 2023; Makel et al., 2012; Schooler, 2014). This crisis was also an important reason to initiate a grant programme for replication studies in the Netherlands. In its call for proposals, the Dutch Research Council wrote:

Replication lies at the heart of the scientific method and makes it possible to build upon previously demonstrated and confirmed scientific findings. Many studies, however, have proved not to be reproducible. If research is not reproducible then this is often attributed to chance, or unintended errors, but p-hacking (i.e., data dredging), publication bias and especially selective reporting will undoubtedly play a major role in this as well. (NWO & ZonMw, 2016, 2017, 2019)

Some of the researcher participants also referred to the replication crisis as a motivation for their project. In their pre-registrations or published papers, they often wrote such things as: ‘In light of the current replication crisis in the social sciences …’ or ‘Given the developed awareness regarding the replication crisis in psychology …’. Some also used the replication crisis as a motivational argument when being interviewed:

We embraced the initiative because we all are supporters of open science and the initiative that emerged in the aftermath of the replication crisis in social psychology. And we’re also concerned after the falsification scandal around Diederik Stapel … all this triggered the initiative by NWO, but also our support for such initiatives. (Lucas)

Additionally, we saw that there are two different forms of motivation for conducting a replication study. First, some researchers wanted to verify a specific result because they suspected that the original study’s result were false. Sven described the original study as being ‘at odds with what all the great theories in the field predict’. Jacob, a methodologist, described the replication target as a typical example of a weak study: ‘[It had] all the characteristics that you expect if you’re a meta-researcher, that um, well, lower the replicability. Small N, not pre-registered, flexible analyses, it’s the whole caboodle.’

Second, other replication researchers wanted to confirm or contribute to the robustness of a finding in their field. For example, Nadia said that she wanted to replicate the study ‘to make a really good estimation of the effect’. Nora said she wanted to determine ‘with certainty’ whether the tested finding existed or not. And Lucas explained he wanted to ‘make sure that this result is robust and can be relied on in future research.’ Thus not all our researchers are motivated by doubt regarding the original study but can also be motivated by the wish to strengthen the original result.

It was a requirement of NWO that the original study be an important ‘landmark’ study, which further strengthened the motivation to find definitive evidence for—or at least a good estimation of—its effect. Nadia told us

If you look at the fact that they still, especially in a higher education context, refer to a study that was conducted so many years ago … with eight [participants] per condition, and yes … . Well for us it really makes us think, then it really makes sense to look at it again systematically, in a bigger sample. To see if you really find that again.

According to some of the researchers, replication research was especially important in some fields where it was rarely done—due to factors such as finances, methodological complexity, or a lack of appreciation. Other researchers emphasized that the original studies were often cited but never checked. Some noted that while several replications existed, they were conducted poorly, or with insufficient participants, and therefore failed to provide sufficient answers. In these situations, replication studies were undertaken to resolve the discussions about numerous small, often conceptual, replications yielding diverse results. Nora and Theo said

Everyone uses different tasks to measure the concepts. And measuring it differently means that you find different things. But … some do find it, some don’t, some find it partially, or don’t find it partially. So, it’s a big mess. And that’s why we decided to do this. (Nora)

Nobody gets it replicated really well. … Everyone used a slightly different paradigm and could never replicate that finding. And that’s why we have decided with exactly the same instruments, eh to replicate that effect. (Theo)

Thus, although replications are not uncommon in some fields of psychology, well-powered and direct—whether exact or ‘mere’—replications remain rare, as Mulkay and Gilbert (1986) observed in biochemistry. The replicators we interviewed see direct replications as important and believe that they contribute to reliable and robust evidence.

## How exact is an exact replication?

As we noted in our introduction, psychologists see direct replication as a good scientific practice to verify or falsify scientific claims. Similarly, the replication researchers we interviewed gave comparable motivations for conducting their studies. However, as pointed out by the sociologists of science in the 1970s and 1980s, two studies are never identical. The funding agency for the replication studies discussed here also struggled with the concept of exact replication. It defined ‘exact’ to be ‘as precise as possible’ and asked the applicants to state why changes are necessary when ‘the design of the research deviates from or has been modified with respect to the original research’. On the other hand, it also demanded a sample size that is ‘large enough’ for the proposed research (NWO & ZonMw, 2016, 2017, 2019). That is, according to the funding call, the exactness of an exact replication was open for discussion, with particular emphasis on the design of the study, to allow methodological improvement.^5^

When we asked replication researchers about differences between the old and the new study, many replicators started by saying that it was ‘exactly’ the same as the original study, or ‘as exact as possible’. However, later in the interviews, all participants identified differences—often improvements over the originals. The language of instructions and stimulus material was often different, new software programmes or other technologies were used, some inclusion criteria (e.g., about health or medication) were adapted, sometimes other population criteria (e.g., different students or no students) were used, and so forth. But all these small changes were seen as justified. Alex listed numerous changes from the original protocol, only to conclude, ‘so we didn’t change anything that would take it away from a direct replication.’

Jacob, a methodologist experienced in replication studies, said that it is simply impossible to do the same experiment after many years. Like Alex, Jacob said that it was about replicating the relevant factors of the experiment:

So that you try to do it as exact as possible. Ehm, of course that is, you can try, but of course it will never go down that way entirely. So, you can never replicate exactly because it’s … 18 years later, well that’s a gigantic difference in time and so on and so on, so ehm, well you’ll never get close to that. And then in the end you’ll have to think a bit more theoretically about are these really relevant factors, yes, or no?

However, what is ‘really relevant’ is not always easy to replicate. For example, David referred to what we might call the historicity of the phenomenon, explaining that the study he wanted to replicate had become so famous that most people were familiar with the hypothesis and results. This ‘foreknowledge’ could affect the results of their study (e.g., because participants could respond more willingly or reluctantly) but it was not something that they could influence:

[T]his study for example has had a lot of media attention. And nowadays everyone knows what [the outcome of the original study was]. Ehm, yes, you can’t really do that replication completely exact anymore. It also has to do with the fact that we cannot influence the foreknowledge of the participant.

Mona, another methodologist with considerable experience in replication studies, acknowledged that she had to modify many aspects of the experiment to make it feasible. In her case, the material of the experiment (a fake journal article) was outdated and had to be remade. ‘It’s always tricky, we do try of course to do a direct replication, but … we have to change quite a lot of things to make it possible at all.’ This was also the situation for Kate, who had to make new videos because the old ones were ‘comical’ rather than stressful nowadays, and for John, who said that the original material gave ‘a bit of a sixties feeling’.

While discussing the differences between the original study and the replication, researchers would refer to different aspects. In some studies, the sameness was achieved through the material or setting (e.g., same tasks, same stimuli), whereas in others, recreating the original stimuli effect required different stimulus material. Moreover, replication researchers who used technologies had to decide if they would use the old technologies, new technologies, or maybe even a combination of both. Mostly, they chose newer instruments—as did Nora and Celine, who were both aware that this could affect the results.

So, our eye tracker is also newer than their version, and the only difference is that ours takes more measurements. That one transmits more signals, so you actually have more reliable data than they had. (Nora, Interview 1)

If we don’t find it, it’s okay. Then we won’t know whether it’s because of that. Perhaps we would find it with fewer measurements, but then we would have to take out data, and that’s not what we want. (Nora, Interview 2)

We replicated as much as we could. But we did use a modern pre-processing pipeline to do that. We used like slightly newer tools that used slightly different algorithms. … And obviously, the scanner that we use is different than the scanner that the original study used. (Celine)

In some cases, these newer methods changed the results. Celine and Nancy concluded that one of the original hypotheses could be replicated with the old method, but not with their newer ‘good practice approach’. John, who conducted his replication using both old and new technologies, concluded that the technology affected the results: ‘[The old technology] had a huge impact … because the eye becomes very light.’ In these cases, newer technology, tools, or analysis methods meant that the replication was not exact, yet the replicators were convinced that these adaptations would provide more reliable results. These researchers allowed a change in the methods to better test the assumed effect.

The replicators we studied acknowledged that no two experiments are ever truly identical, as Collins and colleagues pointed out; nonetheless, they presented their studies as direct or exact replications. Considerations regarding sameness and difference could be focused on three aspects of the experiment. Sometimes the replicators focused on the methods and design, which had to be as close as possible to the original. In other cases, the issue of similarity and difference revolved around the experimental manipulation; as a result, replicators modified the stimulus material because the historical context meant that the old material had acquired a new, incorrect meaning. Finally, in some cases, researchers employed updated methods and instruments, believing that they offer greater precision in measuring the dependent variable than the old ones.

## The algorithmic model: Follow the protocol

According to Collins, attempts at replication and the value placed on them are wrongly informed by the idea that researchers should be able to reproduce a study just by following a set of instructions. These instructions can be found in the method sections, in papers, protocols, or pre-registrations. In our interviews and observations, we found that replicators indeed had such an impression at the beginning of their replication experiment. They expected that conducting a replication would simply involve following the instructions of the original experiment.

For example, Nadia thought that doing a replication would be more straightforward than other types of research: ‘I thought, if you’re going to replicate a study, there’s a lot there already. … I thought it would be clearer than [ordinary research].’ Another replicator, Nora, also thought that the replication experiment would be very easy to conduct, because all the instructions and tasks were available in a computer programme. ‘The only thing that we have had to do was transfer them to a new version of [the software].’ Susan also felt that replicating an experiment is like following a protocol: ‘You can’t adapt anything. You just have to do it by the letter. So, there is no room for any creativity or anything like that. It is really just doing it completely by the book.’ And Sven said, ‘We just follow their procedure.’

However, in practice, it became clear that conducting the replication was not so simple. Nadia struggled with the vague formulations of her predecessor (e.g., ‘Is this an operation or not?’), and Susan similarly found that instructions were not always clearly articulated in the original protocol or paper. ‘Every time I read it, I read something different.’ To find out all the details of the original study, many replicators had to consult the original authors. Even Celine, who thought that the original paper was a very good report, missed some important details:

The paper that we replicate was pretty good in reporting a lot of details. But some were missing. Like, without talking to the authors, we would have not been able to replicate the analysis at all. And that is not because the paper was bad, it was just because it is really difficult to really write down all of the details.

The replication researchers in our study had an algorithmic model of research in mind—assuming that simply following the original protocol would suffice—but this approach required clarification from the original authors regarding specific methodological details.

While preparing and conducting their replication, researchers spent a lot of time and effort making their work accessible to other researchers (e.g., putting all details on open science platforms like OSF). As Susan summed up, ‘trying to be very thorough, publish things online, make a logbook’. The idea behind sharing all materials, procedures, and data is that it facilitates future research, replication, or checking of the results. Jacob said, ‘We share everything, so others can replicate that again.’ Such open science practices represent an algorithmic model of science. For example, Rachel explained that being transparent allows other people to ‘exactly see’ your research decisions:

I also hope that the way we try to set it up so transparently—also with respect to coding and so on—that if people … want to study this process, that we enable that much better because you see exactly what our decision rules were, for the instructions as well as for the coding.

For Theo, it was also important to record the protocol precisely so that others could follow it: ‘Yes, we wrote down a lot of protocols in detail and of course those have to be followed. … So we really literally specified everything.’ Miles anticipated a future in which open science allows us to use and check each other’s work:

Oh, my heaven is that everything is open, so including the review reports, that is not the case now; all the data, all the materials, and the code, so that people can easily build on that, can check easily, yes.

Being transparent, writing everything down exactly, and sharing your work should allow others to continue, replicate, and check it. Some of the replicators seemed to realize that this algorithmic model in open science is a bit utopian. For example, Celine said, ‘It became very obvious that it was very difficult to write this, describe an analysis like this in a way that is easily replicable by somebody.’ Alice taught her students about methodology by having them replicate some of her previous studies, which they usually could not do. ‘So, that is also a nice lesson for me’, she reflected. And Nadia said

We did it like this [and] we want to register that too. Also, with pictures of materials, for example. You can’t always capture it in words. That is kind of … I like that. That someone comes to you, and you say: ‘Well, here’s our material, bye!’ But then I think: ‘Who will do that? It’s so much.’ You need to be really motivated to [get into all this] … I find that difficult.

Moreover, some replicators experienced that protocols that are written today—in times of open science—do not automatically stand on their own. Anton worked as a lab manager on several multi-laboratory replication studies, and he told us that the protocols for these studies sometimes needed clarifications:

I act like the coordinator or in-between person between the protocol owners and my research assistants. So, I help them to understand and translate and do the stuff. … And when we have things, I always contact … the protocol writers, authors. And I ask them. For the current one that we are collecting data, for example, we even had a meeting like this, a Zoom meeting. We went through the protocol and asked them some clarifications. So, either in writing or video conferencing, we communicate and get that kind of information.

Our interviews and fieldnotes thus confirm the observations of Collins and others: Many scientists initially have an algorithmic model of replication research in their mind, but this does not work in practice. We found that replication researchers in psychology initially assumed that original papers and protocols—as well as their protocols and datasets—would be clear enough to follow, check, and replicate. It was primarily through the process of attempting to replicate the experiment that they subsequently noticed that not everything was as clear in a protocol as it had originally seemed. Reading a paper multiple times could give multiple interpretations, and the original authors often needed to clarify the intended meaning. Moreover, our findings show that the limitations of the algorithmic model of replication reappeared in the researcher’s work: Replicators invested considerable effort in writing and sharing exactly what they did; however, they sometimes acknowledged that photographs, video conferences, or other communications were necessary for clarification. Nevertheless, the algorithmic model seems to function as an ideal for replication researchers, which must be approached as closely as possible in practice.

## The enculturational model: Tacit knowledge

According to H. M. Collins (1985), the algorithmic model of science should be replaced by the enculturational model of science: Becoming a good researcher is more like becoming part of a culture than just following instructions. Not all knowledge can be explicated, and there are skills that you can only learn by experience, or by seeing others do the trick. More generally, how to follow rules can never be fully explicated and always involves some degree of tacit knowledge. As noted in the previous section, instructions were at times clarified through photographs and other forms of communication.

We also observed other examples of tacit knowledge, especially in the training of experimenters. For example, Sabine, a research assistant who was being trained as an experimenter, had to learn to act in a ‘strict and cold’ manner to stress out the research participants. She learned this by practising and copying the behaviours of her supervisor:

Eh, at first [it was difficult]. But at a certain point, after a few participants, you have your ways, eh … you find your way in that. And of course, because I had Susan as an example, I tried to copy that a bit. And having an example helps, I think, and practice. I did practice first before I started.

Susan, Sabine’s supervisor, added that the experience of being a research participant was also helpful in becoming a good stress-inducing experimenter:

What I like to do is have trainees be participants as well. So that they see the full procedure as well. That is also helpful, so that you understand what the participant is going through. How it feels. So that everyone who stresses people has also been stressed. So that they know exactly what to feel.

Nadia also used experience and feelings to train a research assistant to become a good experimenter, a warm and gentle experimenter:

Looking at earlier videos [of the experiment] I thought: ‘[She does that] so nice and calm. I should do that too.’ It’s also your own judgment. Which is very difficult for a replication. But it is in you. Go back to that feeling [you had then].

Becoming a strict and cold or a gentle experimenter appears to be something that you must learn by training, experiencing, and feeling. Even with thorough training, these forms of knowledge transmission can be difficult. Nora told us that she preferred to do all the experiments herself because ‘it is very complex, with the eye tracker and so on. It is very sensitive research. … Students sometimes make decisions that are just not tactful.’ The complexity was explicated when Nora had to train a research assistant nonetheless, as she could not do all the experiments herself. Nora gave Jill the following advice:

Takes practice, the eye tracker. Maybe you can find a roommate to practice on. You have to get a feeling for it. … You have my phone number, and those of M and Z, [if you don’t succeed]. [But sometimes you have to] try a few times. It’s the most important data of the study. [You have to] get a feeling for it. You have to have confidence in yourself. If you’re calm, so is your participant. If you’re like shit shit shit, then it will be stressful.

Tracking someone’s eyes requires confidence, calmness, repetition, practice, and in case of an emergency, there are three different people you can call. This sounds rather stressful, and not something that you can learn by reading a text.

Like Collins, we observed that tacit knowledge played a role in experimental practice. In each of the psychological studies where we observed work in the laboratory, the importance of tacit knowledge and skill was obvious, especially in the training of experimenters. Nadia, Susan, Nora, and Jill all needed training, experience, judgment, and feeling to create the essential aspects of the experiment (e.g., stress, friendliness, and well-performed eye tracking).

## The experimenters’ regress

Collins pointed out the existence of what he called the experimenters’ regress: Judging whether an experiment was competently conducted requires knowing the expected results, yet knowing the expected results requires a competent experiment. In our fieldwork, we noticed that replication researchers—although probably not familiar with Collins’s ideas (see, Nosek et al., 2012)—were aware of this problem. For example, Celine said in an interview, ‘There could always be two reasons for [a non-replication], right? [Either] you didn’t do it exactly like they did. [Or …] the effect in the original study was essentially found by chance.’

For many replication researchers, pre-registration of the research plans served to specify pragmatically what counted as replication, and what as replicated results. As Miles explained, ‘If you want to do it well, you will have to record in advance when you think your study is replicated successfully or not. [Otherwise…] people will … always interpret it in their own way.’ Making decisions in advance serves as a pragmatic approach to manage confirmation bias—the tendency to interpret results so that they confirm your ideas—and to address the experimenters’ regress. Surely, researchers can never know what they do not know. But they can decide beforehand about their procedures and results, ask for approval from the experts/original authors, write this all down, and ultimately follow up on any decisions taken. This is basically the idea of pre-registration: Pre-register what you will do, define what will count as a confirmation or rejection, and then act accordingly when the results come in. Ideally, you seek approval from other experts—for example, through a pre-registered report or expert consultation. Then, when you experiment according to your pre-registered procedures and decisions, the answer is predefined. Nadia hoped this would avoid difficult discussions:

First, we try to reproduce the whole procedure as exactly as possible. Based on the information that we have. And we do that close, as close as possible. And so, then the discussion is, before we have the results, what does it mean—when is it a replication, and what does it mean if we don’t find the same results? And that’s what we’re trying to get clear now before we have the results. To prevent all sorts of painful, difficult conversations afterwards.

That is, our replication researchers used pre-registration not only as a method to prevent HARKing—hypothesizing after the results are known (Kerr, 1998)—but also to avoid difficult discussions about how exact a replication was and how its results should be interpreted. These pre-registrations are often shared with original authors or other experts for approval. For example, Sven sent his pre-registration plan to the original authors to give them ‘the chance to respond’.

## Replication and negotiation

Collins wrote that replication results are always based on negotiations. He argued that since the experimenters’ regress cannot be broken empirically, then it must be solved socially. In our fieldwork, we saw many negotiations happening during replication efforts. As described above, even during the set-up phase, there were many interactions with the original author or other experts regarding what could or should be changed and what the best instructions were. Interestingly, we found that most of the discussions about results happened before the data collection began and not—as Collins had found—after the results were obtained. We found that such discussions happened primarily within the core replication team—but also with original researchers and other experts—when writing the pre-registration, discussing the mock data, and deciding on the analysis plan. For example, Nadia explained that they had many discussions about the outcomes in the pre-registration phase:

But when do you have … when do you say: ‘Well, that effect is not so strong after all.’ When do you say: ‘Yes, see, now we have provided really good evidence for that effect ….’ And if you have three outcome measures, what do you do if you find it on one outcome measure, and not on the other?

Nadia and her team solved this issue by initiating an ‘expert meeting’ with several researchers—an original researcher, a statistician, and some experts in the specific field—to decide on these and a variety of other questions.

In Miles’s project, the outcome measure was also a point of discussion. The original study reported a high success rate of 98% (i.e., the percentage of participants who behaved according to the hypothesized criteria). Miles proposed to redo the experiment by taking a 75% success rate as the minimum threshold for a successful replication. Other team members complained that this number was ‘arbitrary’. One of them proposed a 50% success rate, but Miles warned that reviewers might find this criterion ‘too lenient’. After several arguments and calculations to decide between the 75% and 50% success threshold, the proponent of 50% gave in by saying, ‘We need to move forward, so if most of us think reviewers will be happier with additional criteria for replication, then I am happy to go along with the 75%.’

We found that there were many discussions about the results—with team members, experts, original authors, and potential future reviewers—but the majority of these took place before data analysis. Regarding the actual recorded results (i.e., the data), most replication researchers did not want to negotiate. They made remarks such as ‘data are what they are’ (John), ‘let the data speak’ (Alex), and ‘that is out of our hands’ (Nadia). According to Miles, this is what differentiates replication from ordinary research:

But the strange thing is that replication research is completely different from ordinary research. And it doesn’t feel like research. The reason is, with good replication research, you spend months in the preparation, in the pre-registration, if you have all the statistical code, you will need to analyse your data. And then when you have your data, it’s a day’s work, so to speak. Very brief. Because you have everything already.

We noted that replication researchers also applied an algorithmic approach when explaining their approaches in data analysis and interpretation. Indeed, they worked to circumvent the experimenters’ regress by discussing and explicating—pre-registering—what would constitute a successful replication outcome.

However, this approach did not solve all uncertainties for the replicators. Despite all the discussions, decisions, and pre-registrations, some replicators did more analyses than they had originally planned, when it became obvious that the results deviated from the original. From our interview notes:

Yes, we pre-registered everything of course. So, we did everything exactly as it says in the pre-registration. But apart from that we have also done exploratory analyses to see if we couldn’t, one way or another, if we couldn’t show maybe the implication is correct after all, under such and such circumstances.

Thus, some replicators continued with their analyses when they did not find the original effect, to see if there were certain circumstances that might have affected their negative results. Such debates and decisions to conduct additional analyses or steps beyond those originally defined, show that pre-registration does not prevent interpretive flexibility or resolve controversies. More broadly, pre-registration is not a proxy for quality (e.g. Devezer & Penders, 2023).

Moreover, even when there is no discussion about the results, it is not always clear how they should be interpreted. In one multi-lab study the pre-specified criteria implied that the original study’s results had been reproduced in most, but not in all labs. However, according to some members of the replication team, in hindsight, these criteria were too strict or incomplete, as the pre-registered criteria focused only on the final part of the experiment. They argued that due to the similarity of participant behaviour during the experiment, it was in fact a successful replication across all the labs. As replicator Sam told us in an interview, ‘We had to say that it was not replicated, but at the same time the results were strongly in favour of the original study.’ Another team member, Miles, disagreed, stating, ‘We have to follow what we registered, and there it simply says that it wasn’t replicated.’ These researchers agreed on the data and the results, but long debated about the exact phrasing of their publication.

In sum, we saw numerous negotiations regarding the analysis and interpretation of the results. We also saw that a failed replication was not decisive, in the sense that the replicators did not always conclude that the underlying theory was wrong or in doubt. For example, there was sometimes no doubt about the real-world phenomenon that the tested theory aimed to explain. Reflecting on an effect that is often seen with psychiatric patients, Theo concluded, ‘I do think the effect is there, for sure. It is just we cannot show it in the lab.’ Indeed, we repeatedly noticed that this final step—what a failed replication implied for the theory at stake—was rarely discussed, and hardly ever answered. As a result, it appears that replications do not practically determine the existence of a phenomenon, as Collins had presumed.^6^ The meaning of a non-replication was formulated as such by the researchers: ‘It’s an effect that doesn’t generalize well’ (David). ‘It is hard to realize in the real world’ (Theo). ‘It was a chance effect’ (Jacob). ‘If this one particular study does not replicate it, another one may replicate it. So, I don’t see it like a yes or no kind of outcome’ (Anton).

Especially when, as was often the case, the original result was not replicated, researchers were—at least at the time of our study—hesitant to decide what this meant for the theory, or the real-world phenomena. This is interesting in the light of Collins’s conclusion that replications and hence negotiations are necessary to decide about the existence of the phenomenon. In our observations, most replicators seemed to avoid making larger-scale inferences about any real-world phenomena or theories. Instead, they confided the discussion to the results themselves and to the question—we noted that this seemed to be difficult enough in itself—of whether the replication study reproduced the original findings.

## Replication as exploration and reflection

Collins’s work on replication has been very influential, but it has also drawn criticism. One criticism targets Collins’s insistence that psychology must aim at stable, replicable results in order to qualify as science. Recently, Peterson (2015) has argued that the contextual variability of human behaviour makes that an unrealistic aim, an argument put forward long ago by psychologist Gergen (1973). We have already noted that the psychologists in our study were pragmatic with respect to this issue. Although historical and cultural contexts complicated the exactness of some replications, they did not diminish our replication researchers’ aspiration for reproducibility and robustness in psychological effects.

As we noted in the introduction, Collins’s work has also been criticized for its focus on exact replication. For example, Feest (2016, 2019) argues that psychologists in particular should approach replication as exploration of a phenomenon. It was clear that the psychologists we observed did not focus exclusively on direct or exact replication. We already noted that some of them performed additional exploratory analyses in their studies. Moreover, many of them voiced doubts and frustration about the NWO’s strict requirement of an exact replication. Many replication researchers would have liked to do things slightly differently, but this was not allowed by the ‘rules’ of replication. For example, Simon said, ‘If we weren’t under the gun of the NWO replication programme, why would we not vary stuff a bit?’ For David, ‘We strictly replicate, but if I were to do the study myself, yes, maybe I would think about other methods.’ And Boris, who set up a multi-lab study, concluded, ‘You invest a lot, you know, two years, 80 researchers, all those labs following exactly the same protocol. … That you could have run both a direct and a conceptual replication. That would have been nice.’ Many researchers concluded during the replication project that the original protocol was not the best research protocol. ‘What a ridiculous rule’, said Nadia. Doing a direct replication means following other people’s decisions, which are not always perceived as good decisions. As Jacob said, ‘Because it’s a replication, you take what they have done. Then you also inherit the drawbacks.’

However, this does not mean that replication researchers see broader replications—conceptual replications—as superior. They appreciated the special value of direct replications. Sam explained:

Conceptual replications are more similar to normal, standard outcomes, and in my view, they have more chances to get published in journals. … I feel that exact replications need special treatment; they are doing something very valuable. … You want to be sure that the fact that was observed at the beginning is actually there. So, you increase the sample size in a way that is increasing your statistical power.

Miles also thought that direct replications are important because they have different functions than have conceptual replications.

Conceptual replications are regularly seen as more useful, but they have a big drawback. Conceptual replication is especially useful when the original study, if you’re sure that the finding there that it’s true. … The advantage of a direct replication is that you precisely address that original finding. Is it correct, and can we generalize it to another context?

Alex was disappointed when he could not find the right participants to do an exact replication and had to turn his study into a conceptual replication by using students instead of patients. In his view, this made his replication less informative, because it did not lead to a ‘definite answer’. Alex noted, ‘It is not a replication. It is a conceptual replication. It extends on the original study.’ Thus, although replication researchers often made changes—or would have liked to make changes—to the original protocol, they recognized the value of direct replication, as it could ideally lead to definitive answers about the experimental effect. This is quite close to Collins’s aspiration of stability. However, they also felt limited by needing to follow other people’s decisions. Instead, it appeared that the replicators would have preferred to do both direct and conceptual replications. Some of them made efforts in this direction: Celine used old and new analysis methods, John used old and new technologies, David replicated directly and incorporated extensions that aimed to clarify the result, and many other replicators added questionnaires or small tasks to gather extra information.

Thus, our replication researchers were motivated to find out whether a phenomenon exists or not and, at the same time, to explore the phenomenon. Especially Anton, who conducted several replications in multi-lab studies, saw a replication as an exploration:

[A replication] is to experiment. It is to see if this will hold under the circumstances. If it doesn’t, then maybe we have to tweak certain things, we have to change certain variables, we have to change some stuff to better see it. It is like having different glasses to see things in a different environment.

Anton’s different glasses and environment are interesting in light of the historical and cultural context, because his lab is in a country with a slightly different cultural and religious background compared to most other labs. Anton’s motivations go beyond stabilising or exploring a phenomenon, adding an extra layer to the aspiration of replication:

I would say that having this opportunity for researchers around the world to come together, to think about a common problem, and try to offer a solution, I think that is very valuable. So, I look at these replications as opportunities for scientists to communicate and collaborate [e.g., in big team science projects]. Rather than reaching to have final verdict on a theory or a finding.

Anton was proposing to see replications as a means of getting a different perspective and as an opportunity to collaborate. We also observed that conducting replications prompts researchers to think differently. Their negotiations, approaches to the experimenters’ regress, and attempts to make tacit knowledge explicit constitute forms of communication and collaboration.

Moreover, doing replications prompted researchers to reflect on their way of conducting research. For example, Rachel explained:

Being part of this, asking yourself all these things, all those realizations of what the impact is of choices that you make, does influence, for me too, how I now think about the research designs of my own PhD students.

Nora said she learned ‘very different things’ from doing a replication:

You discuss with someone, ‘Actually, why did you use those exclusion criteria?’ That forces you to think about your own research, too. … ‘Huh? Why actually? What’s the purpose of this?’

And David found it ‘inspiring’ for his research to observe how other researchers work: ‘It provides insights into how people think about conducting research and what they consider important in their design and whatnot.’

Replication experiences also affected how researchers read literature or thought about other people’s research. Kate said, ‘It makes you very critical of what you read.’ Nadia felt this also influenced her relationship with her colleagues:

Really, I can’t read an article anymore without thinking: ‘Hey! I miss so much [information].’ … First, you’re not hindered by that knowledge, and now it’s like, you can’t go back. … Then I’m sitting with colleagues and they’re looking at me like: ‘Why are you being so difficult?’ But that difference … [They] haven’t done a replication yet.

John calls the work involved in doing a replication an ‘anthropological kind of research’:

Where did the researcher go wrong, if he did go wrong? That comes more on a detailed level, more biographical level, right? [laughs] What inspired that person? And why did he use this equipment and not that? That kind of thing. So that’s more of a kind of … yeah, anthropological, you could say, kind of research.

That is, we can add to the insights of Collins, Mulkay, Feest, Peterson and others that replication is not only a test of the existence of a phenomenon or an exploration of its historicity and other characteristics, but also a form of collaboration and reflection on how to think about and communicate scientific research and its common challenges.

## Conclusion

We believe that the value of placing our observations alongside those from the replication studies of the 1970s and 1980s does not merely lie in reproducing the ‘finding’ that replication is socially negotiated or involves tacit knowledge. Rather, it lies in how our research highlights the diversity of these negotiations and the broader practices surrounding replication. We have noted several marked differences with the earlier studies. First, the presence of scepticism about the original result in the community was not the only reason to conduct a direct replication. For these psychologists, direct replications are a part of scientific practice, essential for corroborating and exploring earlier findings. Many of the psychologists we interviewed were motivated by a wish to strengthen the original result, measure the effect more precisely, see how well it generalizes to other populations, and so forth. Their attitudes towards the original studies were often not sceptical but neutral.

Second, in our pool of studies, pre-registration and pre-registered reports served an important function in preventing controversies from arising at a later stage. Studies of scientific controversies have identified various ways in which scientific controversies are resolved (Sismondo, 2010), but our material displayed efforts to prevent controversy. We saw much less discussion regarding the theoretical implications of the experiment or the real-world phenomenon the theory aimed to explain. In fact, some of the replicators complained that their replication had little impact on the reputation of the original study, which continued to be cited as factual despite conflicting results (see Hardwicke et al., 2021; Schafmeister, 2021). Despite there having been cases in the last 14 years where replication studies led to discussions about theory and phenomena, in our pool of studies, this was rare. With few exceptions, discussions remained restricted to whether the results replicated those of the original study. For most studies, the ‘decisions about the existence of phenomena’—which, according to H. M. Collins (1985, p. 129), would mark the closure of a replication debate—had not been reached, nor were they actively sought.

Third, our psychologists were aware of the fact that two experiments are never entirely the same. They often introduced changes to the original protocol to improve the study, while still maintaining that it was a direct replication. They expressed exasperation with the funder’s and/or the original authors’ insistence on exact replication. They were typically interested in going beyond direct replication and exploring the phenomenon further in conceptual replications. Thus, they seemed to entertain the kind of wider view of replication that Feest and others have emphasized in their critiques of Collins, giving these critiques some empirical support.

Fourth, in some cases, changes to the experimental procedure were intended to offset the effects of the historicity of some psychological phenomena. The psychologists in our study stayed well clear of epistemological discussions related to the historicity and contextual variability of psychological phenomena—at least in our interviews with them. They were aware that exact replication is impossible—sometimes for reasons of historicity—but believed that such issues could largely be solved pragmatically. For example, replicators realized that the original stimulus material no longer had the same meaning or that participants might be aware of the original study and its results. In such cases, material changes were introduced to recreate the original meaning of the experiment.

Finally, returning to the debate about reflexivity that we briefly discussed in the introduction, we note that our researchers valued replication as an occasion for introspection and reflection, rather than reflexivity. In a contribution to the debate about reflexivity, Woolgar (1988) made a distinction between reflexivity and what he called ‘benign introspection’ (p. 22). Reflexivity, he argued, starts from the assumption that both sociologists of scientific knowledge and the scientists they study are engaged in a similar kind of work: doing research. However, Woolgar noted, scientists are not concerned with reflexivity and its paradoxes because they assume a distinction between themselves and their object of study. Instead, they sometimes engage in benign introspection, ‘following loose injunctions to “think about what we’re doing”’, an activity that Woolgar (1988, p. 22) thought could be ‘perhaps more accurately designated “reflection”’. Our ethnography shows that, at least amongst the psychologists that we studied, replication encourages and necessitates such introspection and reflection among scientists on ‘what they’re doing’. We showed that the ostensibly simple task of repeating the steps of other researchers’ procedures prompted reflection on several issues: what should count as the same experiment and result; the role of tacit knowledge (e.g., skill, judgment, feeling) in science; and the nature and value of replication itself. On a concrete level, each replication is a reflection on the original study, but the scope often widens to encompass science in a broader sense. Replicators’ experiences encouraged them to rethink their research designs and purposes, and to adopt a more critical perspective about both the literature and the research of their colleagues. As Nadia, one of our psychologists, remarked, it stimulated them to ‘reflect a bit on [their] own way of doing science’.

Although Woolgar’s term ‘benign introspection’ may suggest a somewhat superficial process, we believe that such a modest reflection serves to foreground the often-messy process behind the facts and findings. Indeed, the replicators initially believed in the algorithmic model of replication, but they were soon confronted with and reflected on its fundamental shortcomings. While these reflections were often informed by broader discussions on replication and open science in psychology, the concrete work of setting up, conducting, and analyzing a replication experiment was the main impetus. Woolgar (1988, p.14) wrote that ‘reflexivity is the ethnographer of the text’, meaning that reflexivity involves interrogating and revealing the processes that produce a text while being written. In a similar vein, we propose that replication is an ethnography of science, a reflection by the scientists themselves on the scientific process and the challenges of producing results, while being engaged in that very process.

## Notes

1. Feest, correctly in our view, insists on distinguishing these two issues. The first—that reasons rely on tacit knowledge—is a general feature of how concepts are applied. The second—the experimenters’ regress—is specific to situations of uncertainty, in which there is no agreement on how to apply concepts, or whether they are applicable at all.

2. See https://www.nwo.nl/en/researchprogrammes/replication-studies for the replication research programme, and Derksen et al. (2025), and https://replicationinaction.blog/ for more information about the broader project studying projects in this programme.

3. Most psychology interviews were conducted, recorded, and transcribed by JB; five were conducted by MD. For three replication projects, SM conducted the PI and other interviews, as well as one observation, and this material is mainly used for another (forthcoming) article.

4. Formal ethical approval for the ‘Replication in action’ project was waived by the ethics committee of Amsterdam (UMC). We follow the (ABV) ethical guidelines. For more information, see: https://antropologen.nl/abv/ethical-guidelines/.

5. The funding call made some small changes in between the rounds, but we did not see clear effects of this in our interviews. Our quotations from the funding call are the same in the three calls (2016; 2018; 2019). See our blog https://replicationinaction.blog/2022/06/01/how-funding-shapes-research-in-practice/ for more information about the funder and how this might have impacted the replication projects.

6. The Editor of this journal pointed out that this can be framed in terms of interpretive flexibility, and that the meaning of particular experiments can remain open for discussion—a very Collinsish point.
